# Health Insurance Coverage and Its Impact on Medical Cost: Observations from the Floating Population in China

**DOI:** 10.1371/journal.pone.0111555

**Published:** 2014-11-11

**Authors:** Yinjun Zhao, Bowei Kang, Yawen Liu, Yichong Li, Guoqing Shi, Tao Shen, Yong Jiang, Mei Zhang, Maigeng Zhou, Limin Wang

**Affiliations:** 1 Department of Risk Factor Surveillance, National Center for Chronic and Non-communicable Disease Control and Prevention, Beijing, China; 2 Actuarial Department, China Life Insurance Company Limited, Beijing, China; 3 School of Statistics, University of International Business and Economics, Beijing, China; 4 Chinese Field Epidemiology Training Program Department of Education and Training, Chinese Center for Disease Control and Prevention, Beijing, China; College of Veterinary Medicine, China

## Abstract

**Background:**

China has the world's largest floating (migrant) population, which has characteristics largely different from the rest of the population. Our goal is to study health insurance coverage and its impact on medical cost for this population.

**Methods:**

A telephone survey was conducted in 2012. 644 subjects were surveyed. Univariate and multivariate analysis were conducted on insurance coverage and medical cost.

**Results:**

82.2% of the surveyed subjects were covered by basic insurance at hometowns with hukou or at residences. Subjects' characteristics including age, education, occupation, and presence of chronic diseases were associated with insurance coverage. After controlling for confounders, insurance coverage was not significantly associated with gross or out-of-pocket medical cost.

**Conclusion:**

For the floating population, health insurance coverage needs to be improved. Policy interventions are needed so that health insurance can have a more effective protective effect on cost.

## Background

In the past three decades, China has experienced the largest human migration in history, leading to a dramatic rise in the urban population from 191 million in 1980 to 622 million in 2009 – an increase driven largely by rural-to-urban migration [1]. China enforces the “hukou” system, which is a household registration operated by the central government. Its record is issued per family and usually includes births, deaths, marriages, and moves of all members in the family. The hukou system is classified as urban hukou status and rural hukou status, and it is difficult for Chinese citizens to transit from rural hukou status into urban hukou status. China's social welfare is closely tied to the status in hukou system [2]. The rural-to-urban migration coupled with the hukou system have created the unique floating population, which consists of those residing at their residences without hukou (which differ from their hometowns with hukou) for at least six months within a specific year. It is estimated that nearly 40% of the people living in the urban areas are floating population, leading to a total number of about 260 million [3]. In China, many social welfare and healthcare benefits are localized, i.e., only available to those with hukou at their current residences, making them inaccessible to the floating population [4], [5]. The floating population usually has low socioeconomic status and is less-advantaged. Their wellbeing has drawn significant recent attention from the government, social media, press and social and public health researchers [3]. This study focuses on health insurance and its impact on medical cost for the floating population.

The current health insurance system in China is composed of basic health insurance and commercial health insurance. The basic health insurance consists of three schemes for different groups of people and takes different forms in rural and urban areas [6]. The rural areas are covered by the New Rural Cooperative Medical Scheme (NCMS). The urban areas are covered by the Urban Employee Basic Medical Insurance (UEBMI, which is for the employed) and Urban Resident Basic Medical Insurance (URBMI, which covers the unemployed, children, and elderly). Combined together, the basic health insurance covers 95% of the total population [6]. Multiple studies have demonstrated the overall high coverage and effectiveness of the basic health insurance system in China [6], [7]. However, such results are not necessarily applicable to the floating population. Access to healthcare and utilization of health insurance for the floating population face barriers. First, the hukou system poses certain constraints. In the floating population, most have their hometowns with hukou in rural areas while their residences are in urban areas. Hukou is not easily transferable from rural to urban areas. The current basic health insurance system is tightly tied to hukou, and the floating population from rural areas has been explicitly excluded from the urban health insurance until very recently [8]. In principle, the employed floating population in urban areas can be covered by health insurance provided by their employers under UEBMI. However, employers usually lack motivation or pressure to do so [9]. Second, the current health insurance system lacks flexibility especially portability. Because of localized management, health insurance was not transferable until 2009, when major healthcare and health insurance reform took place in China [6]. In 2009, the central government implemented two policies to guide the transfer of health insurance (from hometowns with hukou to residences) for the floating population [10]. The new policy stated that when moving to another residence, the floating population could transfer the individual health insurance account accumulation but lose the accumulation from employers' contribution to the social pooling account. Since the floating population is usually highly mobile, with the risk of losing the accumulation from employers' contribution, the floating population is unlikely to participate in health insurance at residences [11]. Third, the current healthcare system may also prevent the floating population from using insurance at hometowns with hukou. For the aforementioned reasons, the floating population is more likely to have health insurance at hometowns with hukou. By law, the floating population migrating from rural to urban areas should be covered by NCMS at hometowns with hukou. However, to utilize health insurance, the insured are required to have medical services at hometowns. With their general low socioeconomic status, it is highly unlikely that the floating population can go back to hometowns for medical services on a regular basis. As a result, many members of the floating population have chosen to not participate in basic insurance [11]. With the aforementioned barriers, health insurance coverage of the floating population has lagged far behind the rest of the population [11].

Although a large number of studies have been conducted on China's health insurance, our literature review suggests that research on the floating population's insurance remains sparse, especially on a micro level. Notable studies include one by Giles et al. [11], which reviewed the history of social insurance policy and coverage in urban China, documented the evolution of pension coverage and medical and unemployment insurance for both local residents and migrants (from rural areas), and highlighted the obstacles in expanding coverage. Gong and others [3] suggested that migrant workers consistently underused health services both at their hometowns with hukou and at residences. A large study in Shenzhen, a large city hosting a significant number of migrant workers, showed that 55% of the migrant workers were uninsured, and 62% of those who reported illness did not seek professional care [12]. Lin and others [9] examined the determinants of social insurance participation among the floating population, from three different perspectives (government, employer, and floating population), using data from a survey conducted in the Fujian province in 2006. A survey, conducted in Zhejiang province in 2004, explored the living and working conditions, health status, and healthcare access of the floating population and compared against those of the rural and urban residents with hukou [13]. Mou et al. [12] found that health insurance resources inequitably distributed among migrant workers, and that women who were younger, less-educated, and lower paid were more likely to be uninsured and therefore have to pay a significant amount of out-of-pocket cost for healthcare.

This study has been motivated by the following considerations. First, although in the literature there are many studies on health insurance coverage and medical expenditure in China, research on the floating population remains limited. China is experiencing fast urbanization. Studying the floating population, which are vulnerable to illness and its consequences but have been much ignored, can be valuable. Second, the existing studies on the floating population's insurance have been mostly conducted on a macro level, with insufficient attention on personal behaviors and outcomes, and been limited to certain areas. There is a need for a micro analysis on the national level. Third, the existing studies are also limited in that they have often focused on specific type of insurance, for example NCMS only or insurance either at hometowns with hukou or residences. From the end-users' perspective, it is more sensible to consider multiple types of health insurance and health insurance at hometowns with hukou and residences. Last but not least, with the fast development and system-wide reform started in 2009, observations made in earlier studies may not hold [14]. There is a need for an updated description.

This study has two main objectives. The first is to study insurance coverage and identify the associated subjects' characteristics, which is helpful to identify the features of people without insurance. The second is to investigate the association between insurance coverage and medical cost, which is beneficial to estimate the impact of insurance coverage on medical cost. To this end, we conducted a survey study and collected and analyzed micro data. This study is among the few that focus on insurance coverage and its impact for the floating population in China and may provide valuable insights beyond the existing studies.

## Method

### Data Collection

In 2012, the National Center for Chronic and Non-communicable Disease Control and Prevention (NCNCD) conducted a national survey study on chronic diseases and risk behavioral factors of the floating population [15]. A total of 51,000 individuals were scheduled to be sampled, among whom 48,052 completed the survey. In this study, a telephone survey was conducted on 3,000 randomly sampled subjects registered with phone numbers, with the 2012 survey study as the sampling frame. At the beginning of each study, interviewer introduced the purpose of this study, the nature of questions to be asked and asked the participant whether he\she agreed to participant the study. Once the participant provided his\her verbal informed consent to participate this study, the survey continued. We promised to all participants that all their information collected can only be used in this scientific study and will not open to public. All verbal participants' informed consents are saved in the phone call record. Since the survey was conducted by telephone instead of by face to face interview, it is difficult to ask participants to provide written informed consent. This study including the consent procedure was approved by the research ethics review committee at Chinese Center for Disease Control and Prevention (China CDC).

In sample selection, stratification by area and region was considered in an attempt to achieve representativeness. The resulted samples were from 32 provinces and municipalities. Among the 3,000 phone numbers, 1,657 were not eligible, including those associated with non-floating population, disconnections, and wrong numbers. The remaining 1,343 subjects included those fully interviewed, partially interviewed, eligible but not interviewed (refusal after eligible subjects identification (ESI), language or communication barriers after ESI, and termination before finishing the questionnaires), and with unknown eligibility (busy line, no answer, phone no longer in service, language or communication barriers before ESI, hang-up or termination before ESI). Ultimately, 644 subjects completed the interview, with a response rate of 48.0%.

Two categories of data were collected. The first were on demographic and personal characteristics, including gender, age, marital status, education, occupation, personal income, and personal expenditure. Information on whether a subject was covered by health insurance and insurance types both at hometowns with hukou and residences was collected. As our pilot study suggested a very small number of inpatient treatments, we collected data on the presence of chronic diseases as a surrogate of health condition. The second category of data was on medical cost particularly including both gross and out-of-pocket (OOP) medical cost. The gross cost was defined as the accumulated medical cost during a period of twelve months prior to the survey. The OOP cost was defined as the gross cost minus insurance reimbursement.

The dateset now is password-protected and securely stored in China CDC. Before we got participants' informed consent, we promised to all participants that all their information collected can only be used in this scientific study and will not open to public. Also per funding regulations, the dataset can not be publicly available. Access to that data needs to be applied and approved on a case-by-case basis.

### Statistical Analysis

Summary statistics were first computed for the whole cohort. We then compared subjects with insurance coverage against those without. Insurances at hometowns with hukou and at residences were analyzed separately. As the variables are categorical, p-values were calculated using Chi-squared tests. Multivariate analyses were then conducted, searching for factors associated with insurance coverage. As the response variable is binary, logistic regressions were conducted. The adjusted odds ratios (aOR) and p-values were computed. In the analysis of medical cost, gross and OOP costs were analyzed separately. In previous study, medical cost has been categorized every 1K RMB [6]. We first contrasted those with gross cost over 2K RMB (integer number close to the third quartile) against those with a lower cost. In the analysis of OOP cost, the two groups had over 0.5K RMB cost (integer number close to the third quartile) and lower than 0.5K RMB cost. Chi-squared tests were conducted, and p-values were computed. Multivariate regression analyses on cost were then conducted. For binary response variables, logistic models were adopted, and aOR and p-values were calculated. In the analysis of cost, the role of insurance coverage was of special interest. Analysis was conducted using SAS Version 9.3 (SAS Software Inc.).

## Results

### Characteristics of coverage

Among the surveyed subjects, at their hometowns with hukou, 63.4% and 2.2% had basic and commercial insurance, respectively, and 34.5% had no insurance. At their residences, 31.9% and 3.6% were covered by basic and commercial insurance, respectively, and 64.9% had no insurance. In total, 82.2% of the subjects were covered by basic insurance at hometowns with hukou or residences. The commercial insurance coverage rate was 5.1%. The UEBMI coverage rate at residences (26.2%) was higher than that at hometowns with hukou (11.7%), while the URBMI and NCMS coverage rates at residences (3.7% and 2.2%, respectively) were lower than those at hometowns with hukou (8.5% and 43.6%, respectively).

Subjects' characteristics are summarized in Table 1. No association with insurance coverage was observed for gender, age, and marital status. The association between education and insurance coverage at residences was significant (p-value <0.001). In general, those with a higher education were more likely to have insurance. For example, for those with insurance at residences, 46% were educated with junior college and more, compared to 26.4% for those without insurance. However, this association was reversed at hometowns with hukou. The association between occupation and coverage at residences was also significant. For example, among those with insurance, 25.1% worked in manufacture, compared to 15.1% among those without insurance. There is a significant association between coverage at hometowns with hukou and the presence of chronic diseases (p-value <0.001). Subjects with chronic diseases were more likely to have insurance. Personal expenditure was significantly positively associated with coverage at residences. No association was observed for personal income.

**Table 1:** Basic characteristics of all subjects and subsets with different insurance coverage status.

Variable	Total	Insurance		Insurance	
		at hometowns with hukou		at residences	
		No	Yes	No	Yes
	586	204(34.8)	382(65.2)	371(63.3)	215(36.7)
**Gender**					
Male	337	110(53.9)	227(59.4)	222(59.8)	115(53.5)
Female	249	94(46.1)	155(40.6)	149(40.2)	100(46.5)
p-value		0.219		0.14	
**Age**					
18–29	208	79(38.7)	129(33.8)	127(34.2)	81(37.7)
30–39	177	57(27.9)	120(31.4)	113(30.5)	64(29.8)
40–49	142	51(25)	91(23.8)	85(22.9)	57(26.5)
50+	59	17(8.3)	42(11)	46(12.4)	13(6)
p-value		0.424		0.129	
**Marital Status**					
Single	135	51(25)	84(22)	88(23.7)	47(21.9)
Married	444	152(74.5)	292(76.4)	279(75.2)	165(76.7)
Divorce/Widowed	7	1(0.5)	6(1.6)	4(1.1)	3(1.4)
p-value		0.517		0.883	
**Education**					
No schooling	12	1(0.5)	11(2.9)	11(3)	1(0.5)
Primary	32	7(3.4)	25(6.5)	25(6.7)	7(3.3)
Junior school	166	40(19.6)	126(33)	125(33.7)	41(19.1)
Senior school	179	66(32.4)	113(29.6)	112(30.2)	67(31.2)
Junior college and more	197	90(44.1)	107(28)	98(26.4)	99(46)
p-value		<0.001		<0.001	
**Occupation**					
Manufacture	110	37(18.1)	73(19.1)	56(15.1)	54(25.1)
Retail	107	29(14.2)	78(20.4)	79(21.3)	28(13)
Hotels and catering	96	37(18.1)	59(15.4)	67(18.1)	29(13.5)
Social Services	110	43(21.1)	67(17.5)	63(17)	47(21.9)
Construction	85	27(13.2)	58(15.2)	62(16.7)	23(10.7)
Others	78	31(15.2)	47(12.3)	44(11.9)	34(15.8)
p-value		0.303		0.002	
**Presence of chronic diseases**					
Yes	474	152(74.5)	322(84.3)	305(82.2)	169(78.6)
No	112	52(25.5)	60(15.7)	66(17.8)	46(21.4)
p-value		<0.001		0.316	
**Personal expenditure**					
Less than 9K	139	48(23.5)	91(23.8)	103(27.8)	36(16.7)
9K–12K	56	15(7.4)	41(10.7)	38(10.2)	18(8.4)
12K–24K	331	118(57.8)	213(55.8)	200(53.9)	131(60.9)
24K and more	60	23(11.3)	37(9.7)	30(8.1)	30(14)
p-value		0.587		0.002	
**Personal income**					
Less than 24K	144	59(28.9)	85(22.3)	91(24.5)	53(24.7)
24K–30K	150	45(22.1)	105(27.5)	91(24.5)	59(27.4)
30K–36K	148	50(24.5)	98(25.7)	93(25.1)	55(25.6)
36K and more	144	50(24.5)	94(24.6)	96(25.9)	48(22.3)
p-value		0.273		0.729	

*Count (percentage).

### Multivariate analysis of coverage rate

The results are presented in Table 2. For insurance coverage at hometowns with hukou, the significantly associated factors include education, occupation, and presence of chronic diseases. Specifically, with no schooling as the baseline, subjects with senior school and junior college and more were significantly less likely to have insurance (aOR 0.105 and 0.071, respectively). With social services as the baseline for occupation, subjects working in hotels and catering were less likely to have insurance (aOR 0.604, p-value 0.069, which is borderline significant), while those working in retail were more likely to have insurance (aOR 1.336, p-value 0.052, which is borderline significant). Subjects with chronic diseases were more likely to have insurance.

**Table 2:** Multivariate logistic regression analysis of insurance coverage at hometowns with hukou and residences.

	Insurance		Insurance	
	at hometowns with hukou		at residences	
	aOR	p-value	aOR	p-value
**Gender(baseline: male)**				
Female	0.709	0.104	1.37	0.136
**Age(baseline:** 18–29**)**				
30–39	1.322	0.462	0.736	0.829
40–49	1.043	0.515	0.889	0.202
50+	1.357	0.558	0.387	0.027
**Education(baseline: No schooling)**				
Primary	0.239	0.739	3.427	0.720
Junior school	0.219	0.853	4	0.991
Senior school	0.105	0.012	6.757	0.057
Junior college and more	0.071	<0.001	10.879	<0.001
**Marital status(baseline: Single)**				
Divorce/Widowed	1.495	0.691	4.58	0.159
Married	0.906	0.608	1.847	0.744
**Occupation(baseline: Social services)**				
Manufacture	0.999	0.543	1.462	0.015
Retail	1.336	0.052	0.519	0.008
Hotels and catering	0.604	0.069	0.836	0.670
Construction	0.872	0.943	0.678	0.196
Others	0.688	0.256	1.391	0.063
**Presence of chronic diseases(baseline: No)**				
Yes	2.04	0.002	0.808	0.374
**Personal expenditure (baseline: Less than 9K)**				
9K–12K	1.674	0.235	1.098	0.150
12K–24K	1.222	0.968	1.633	0.832
24K and more	1.116	0.706	3.496	0.002
**Personal income(baseline: Less than 24K)**				
24K–30K	1.713	0.143	1.004	0.119
30K–36K	1.463	0.599	0.734	0.714
36K and more	1.301	0.862	0.501	0.022

In the analysis of insurance coverage at residences, the significantly associated factors include age, education, occupation, personal expenditure, and personal income. More specifically, with age 18–29 as the baseline, subjects aged 50+ were less likely to have insurance (aOR 0.387, p-value 0.027 borderline significant). With no schooling as the baseline, those with senior school (aOR 6.757, p-value 0.057) and with junior college and more (aOR 10.879, p-value <0.001) were more likely to have insurance. With social services as the baseline for occupation, subjects working in manufacture (aOR 1.462, p-value 0.015), retail (aOR 0.519, p-value 0.008), and others (aOR 1.391, p-value 0.063 borderline significant) had different coverage rates. With less than 9K as the baseline, subjects with personal expenditure of 24K and more had a higher probability of insurance coverage (aOR 3.496, p-value 0.002). With less than 24K as the baseline, those with personal income of 36K and more had a lower probability of insurance coverage (aOR 0.501, p-value 0.022).

### Characteristics of medical cost

The average gross and OOP costs were 1.9K RMB (sd  = 6.1K) and 0.9K RMB (sd  = 3.0K), respectively. The analysis results comparing different cost groups are presented in Table 3. For gross cost, age has a significant association (p-value 0.001). For example for the 2K and more group, 23.3% were in the 18–29 age group, compared to 37.9% for the less than 2K group. A significant association was observed for education. Subjects with a higher education tended to have lower gross cost. A significant association was also observed for the presence of chronic diseases. It is interesting to note that the insurance coverage at hometowns with hukou or residences was not significantly associated with gross cost. For OOP cost, females were more likely to have a higher cost (p-value 0.041). There is a significant association for age. For example, for the 0.5K and more group, 27% aged between 18 and 29, compared to 39.2% for the less than 0.5K group. The association for education is also significant. For example for the 0.5K and more group, 26.3% were educated with junior college and more, compared to 38.6% for the less than 0.5K group. The presence of chronic diseases was significantly associated with a higher cost (p-value <0.001). The association for personal income was borderline significant. Again it was noted that the insurance coverage was not significantly associated with OOP cost.

**Table 3:** Characteristics of subjects with different gross and OOP medical cost status.

	Gross cost		OOP cost	
	Less than 2K	2K and more	Less than 0.5K	0.5K and more
	385(78.9)	103(21.1)	342(71.4)	137(28.6)
**Gender**				
Male	223(57.9)	63(61.2)	209(61.1)	72(52.6)
Female	162(42.1)	40(38.8)	133(38.9)	65(47.4)
p-value	0.656		0.041	
Age				
18–29	146(37.9)	24(23.3)	134(39.2)	37(27)
30–39	122(31.7)	28(27.2)	105(30.7)	42(30.7)
40–49	81(21)	37(35.9)	70(20.5)	45(32.8)
50+	36(9.4)	14(13.6)	33(9.6)	13(9.5)
p-value	0.001		0.013	
**Marital Status**				
Single	92(23.9)	17(16.5)	84(24.6)	26(19)
Married	291(75.6)	84(81.6)	255(74.6)	110(80.3)
Divorce/Widowed	2(0.5)	2(1.9)	3(0.9)	1(0.7)
p-value	0.147		0.261	
**Education**				
No schooling	5(1.3)	5(4.9)	6(1.8)	4(2.9)
Primary	14(3.6)	12(11.7)	10(2.9)	14(10.2)
Junior school	110(28.6)	27(26.2)	95(27.8)	37(27)
Senior school	121(31.4)	28(27.2)	99(28.9)	46(33.6)
Junior college and more	135(35.1)	31(30.1)	132(38.6)	36(26.3)
p-value	<0.001		0.001	
**Occupation**				
Manufacture	72(18.7)	21(20.4)	64(18.7)	27(19.7)
Retail	71(18.4)	21(20.4)	62(18.1)	26(19)
Hotels and catering	63(16.4)	14(13.6)	52(15.2)	26(19)
Social Services	76(19.7)	12(11.7)	66(19.3)	21(15.3)
Construction	50(13)	22(21.4)	46(13.5)	25(18.2)
Others	53(13.8)	13(12.6)	52(15.2)	12(8.8)
p-value	0.164		0.408	
**Presence of chronic diseases**				
Yes	315(81.8)	73(70.9)	287(83.9)	94(68.6)
No	70(18.2)	30(29.1)	55(16.1)	43(31.4)
p-value	0.004		<0.001	
**Personal expenditure**				
less than 9K	87(22.6)	25(24.3)	79(23.1)	33(24.1)
9K–12K	40(10.4)	9(8.7)	40(11.7)	10(7.3)
12K–24K	219(56.9)	58(56.3)	189(55.3)	79(57.7)
24K and more	39(10.1)	11(10.7)	34(9.9)	15(10.9)
p-value	0.950		0.563	
**Personal income**				
Less than 24K	89(23.1)	28(27.2)	75(21.9)	38(27.7)
24K–30K	100(26)	28(27.2)	85(24.9)	43(31.4)
30K–36K	99(25.7)	24(23.3)	94(27.5)	26(19)
36K and more	97(25.2)	23(22.3)	88(25.7)	30(21.9)
p-value	0.797		0.061	
**Insurance at residences**				
No	244(63.4)	65(63.1)	214(62.6)	85(62)
Yes	141(36.6)	38(36.9)	128(37.4)	52(38)
p-value	0.929		0.969	
Insurance at hometowns with hukou				
No	130(33.8)	36(35)	114(33.3)	51(37.2)
Yes	255(66.2)	67(65)	228(66.7)	86(62.8)
p-value	0.982		0.709	

*Count (percentage).

### Multivariate analysis of medical cost

The results are presented in Table 4. For gross cost, the significantly associated factors included age, education, occupation, and presence of chronic diseases. With 18–29 as the baseline, the 40–49 age group had higher cost (aOR 2.455, p-value 0.017). With no schooling as the baseline, all other four groups had lower costs (aOR 0.725, 0.199, 0.193, and 0.209, respectively). With social services as the baseline for occupation, the construction group had significantly higher cost (aOR 3.398, p-value 0.025). Subjects with chronic diseases tended to have lower gross cost (aOR: 0.539, p-value 0.030). For OOP cost, the significantly associated factors include age, education, occupation, presence of chronic diseases, and personal income. A similar observation was made on age as for gross cost. With no schooling as the baseline for education, junior college and more had lower cost (aOR 0.318, p-value 0.004), while primary school had higher cost (aOR 1.876, p-value 0.012). With social services as the baseline for occupation, those working in construction were more likely (aOR 2.293, p-value 0.037) to have higher costs. A similar observation was made on the presence of chronic diseases as for gross cost. With less than 24K as the baseline for personal income, the 24–30K and 30K–36K groups were borderline significantly different. Insurance coverage was not significantly associated with the level of OOP cost.

**Table:** **Table 4.** Multivariate logistic regression analysis of gross and OOP cost.

	Gross cost >2K		OOP cost >0.5K	
	aOR	p-value	aOR	p-value
**Gender(baseline: male)**				
Female	0.75	0.296	1.319	0.262
**Age(baseline:** 18–29**)**				
30–39	1.225	0.364	1.388	0.890
40–49	2.455	0.017	2.216	0.016
50+	1.610	0.783	1.082	0.464
**Education(baseline: No schooling)**				
Primary	0.725	0.056	1.876	0.012
Junior school	0.199	0.023	0.459	0.101
Senior school	0.193	0.017	0.58	0.479
Junior college and more	0.209	0.051	0.318	0.004
**Marital status(baseline: Single)**				
Divorce/Widowed	1.905	0.505	0.619	0.780
Married	0.832	0.382	0.764	0.963
**Occupation(baseline: Social services)**				
Manufacture	2.352	0.397	1.643	0.409
Retail	2.111	0.684	1.25	0.751
Hotels and catering	1.782	0.824	1.837	0.217
Construction	3.398	0.025	2.293	0.037
Others	1.576	0.528	0.699	0.034
**Presence of chronic diseases(baseline: No)**				
Yes	0.539	0.030	0.352	<0.001
**Personal expenditure (baseline: Less than 9K)**				
9K–12K	0.98	0.657	0.779	0.133
12K–24K	1.329	0.407	1.556	0.228
More than 24K	1.267	0.740	2.025	0.129
**Personal income(baseline: Less than 24K)**				
24K–30K	0.896	0.453	1.017	0.067
30K–36K	0.672	0.547	0.486	0.060
36K and more	0.569	0.232	0.537	0.207
**Insurance at hometowns with hukou(baseline: No)**				
Yes	1.098	0.728	1.237	0.395
**Insurance at residences(baseline: No)**				
Yes	0.936	0.807	1.02	0.938

## Discussion and Conclusion

### Main findings

The observed coverage rate of basic insurance – either at hometowns with hukou or residences – was significantly lower than that reported by the central government for the whole population but similar to that reported in [6]. The Chinese government has made significant effort to increase insurance coverage in the recent years and pledged to achieve universal coverage by 2020 [11]. However, as partly described in the Background section, with significant barriers, the insurance coverage rate for the floating population has been lagging behind. The identified lower coverage rates can provide empirical basis for future policy development. It is interesting to observe the differences in coverage between hometowns with hukou and residences. More specifically, lower coverage rates at residences are observed, which accords with the fact that under the current system, it is more difficult for the floating population to obtain and maintain insurance coverage at residences. As the floating population consists of those staying at residences for over six months a year, it is critical that they are properly covered by health insurance at residences. At residences, most insurance was UEBMI, which accords with the fact that the majority of migrants can get themselves covered by basic insurance only through their employers [9]. Improving coverage at residences particularly deserves more attention. The commercial insurance coverage rate was much lower than that reported in [6]. It accords with the fact that commercial insurance mainly aims at the upper class in China, while the floating population overall has a lower socioeconomic status.

Multiple personal characteristics are identified as associated with insurance coverage. It is observed that insurance at hometowns with hukou and at residences follows different patterns. In the literature [5–7,9,12], it has been noted that the pursuit of insurance coverage is a complicated process associated with multiple demographic and personal characteristics. In addition, as described in the Background section, the pursuit of insurance coverage for the floating population faces unique barriers not shared by the rest of the population. It is interesting to note that the estimated effects have “conflicting directions” for insurance at hometowns with hukou and at residences. This can be partly explained by the compensating effects of the insurance coverage at two places. That is, as a subject stays more than six months at residence, if he/she has coverage at residence, it is more likely he/she will not pursue coverage at hometown. For insurance at residences, the more-educated group has a significantly higher aOR than the baseline. Subjects with more education tend to have a higher socioeconomic status [7]. The association between education and pursuit of insurance has been noted in the literature [16], which suggested that failing to understand the insurance system and the complexity of reimbursement process could prevent the less-educated from pursuing insurance. Occupation is also observed to play a role. Different occupations have different degrees of participation and different amounts of contribution. As detailed information on the occupation-specific regulations is not available, it is not completely clear how to interpret the occupational differences. Insurance expenditure is a component of overall personal expenditure. For insurance at residences, subjects with lower personal expenditure and higher personal income are less likely to be covered by insurance. The observed positive association between personal expenditure and insurance coverage at residences is consistent with that in [6]. In terms of socioeconomic status, groups with lower income and lower expenditure are less-advantaged [7]. A possibility for the conflicting association for the income and expenditure is that floating population generally need to send money to their families at hometowns, which plays a significant role in improving their families' wellbeing [17] and thus their personal income may not fully reflect their real economic status.

In the multivariate analyses of gross and OOP medical costs, multiple factors are identified as significant. As has been noted in many published studies, the level of medical cost is determined by health and healthcare status, insurance coverage and utilization, personal characteristics, and others. The association between age and cost has been observed in multiple published studies [6], [7], [16], [18]. Subjects with higher education are more likely to be in lower cost group, which is consistent with [12]. Education is correlated with the overall socioeconomic status, occupation, and other factors. It is observed that those working in construction have significantly higher costs. Compared with other occupations, there is a higher risk of, for example, work-related injuries for construction [19]. The negative association between the presence of chronic diseases and cost can be counterintuitive. It has been observed in the literature that the majority of rural-to-urban migrants have low income and are frequently laid off [20]. Those with chronic diseases are especially less-advantaged, making them less likely to visit doctors and/or spend money treating diseases. Because of resource and design limitations, detailed information on health conditions was not collected. The presence of chronic diseases, although can partly reflect health conditions, is far from comprehensive. More detailed investigation is needed to fully comprehend the association for presence of chronic diseases. Borderline significant associations are observed for personal income, although there is a lack of linearity. As pointed out in the literature [21]–[23], income is also correlated with multiple other factors, which may contribute to the complicated association. A factor unique to the floating population is that they usually need to support family members at hometowns with hukou. Thus, their personal income may not fully represent their economic status. It is interesting to observe insignificant associations for insurance at hometowns with hukou and at residences. An effective insurance system should be able to protect the financial wellbeing of the insured by reducing cost. The lack of significant association can be caused by multiple factors, for example the utilization barriers discussed in Background and problems caused by failing to understand the system. As has been discussed in the literature, overall the coverage depth of basic insurance is still very limited, and the insured in China still end up paying a high amount of medical cost out of pocket. In addition, the floating population has a lower coverage rate than the general population, which further diminishes the protecting effects of insurance.

### Limitations

This study has limitations. The survey collected information for a period of twelve months. For a subject, gross cost and OOP cost may vary from year to year. Although it was possible to collect information for a longer period, such an effort was not pursued because of concerns about possible recall error [7]. The survey collected cross-sectional observational data. With such data, only association, not causality, can be inferred. Many published studies share the same limitation [6], [16], [24]. The nature of survey inevitably led to certain drawbacks, including limited information, possible recall bias, and others [25]. For example, the survey only collected information on whether a subject was covered by insurance. If not, there was no follow-up question on why not covered. Without collecting additional information, we are not able to draw more affirmative conclusions. Another limitation is that there is no detailed information on illness. For example, it will be interesting to see if there is still no association between insurance coverage and medical cost after more appropriately adjusting for illness information. This study may also be limited by having a moderate sample size, especially considering that the subjects were from 32 provinces and China has significant regional variations. There are a number of subjects with very high medical costs. Ideally, stratified analysis should be conducted, investigating the different behaviors for different medical cost groups. However, with a limited sample size, such an effort is not pursued. Since this study was not able to survey those without cell phone, the results may not be applicable to the whole floating population. Additionally, although we have an assumption that non-respondents are totally at random, actually only half of subjects responded to the survey, which may hardly lead to results without bias at all.

## Conclusion

Research on the floating population's healthcare and health insurance is of significant importance. In this article, we report empirical observations made in a survey recently conducted on insurance coverage and medical cost. A great discrepancy of insurance coverage exists between the floating population and the general population. Demographic and personal characteristics are found as associated with insurance coverage. The findings may have important implications and can assist the development of intervention programs to further increase coverage and effect. The analysis of medical cost leads to two main observations. The first is that insurance coverage is not associated with gross and OOP medical costs. The second is the distinct associations with medical cost for the floating population. More detailed investigations are needed to fully understand the mechanisms underlying such associations. More effective strategies are needed to improve coverage depth and reduce financial burden caused by illness.
